# Cognitive Health After Cerebellar Stroke: Potential Link Between Socio-Educational Status and Memory Outcome

**DOI:** 10.1007/s12311-024-01775-x

**Published:** 2025-01-07

**Authors:** Philippe Voruz, Ioana Medeleine Constantin, Emilie Chassot, Marine Thomasson, Frédéric Assal, Julie Anne Péron

**Affiliations:** 1 Clinical and Experimental Neuropsychology Laboratory, Faculty of Psychology, Geneva, Switzerland; 2 Neurosurgery Department, Geneva University Hospitals and Faculty of Medicine, Geneva, Switzerland; 3 Neurology Department, Geneva University Hospitals and Faculty of Medicine, Geneva, Switzerland; 4 Faculty of Psychology and Educational Sciences, 40 bd du Pont d’Arve, Geneva, 1205 Switzerland

**Keywords:** Cerebellum, Memory, Neuropsychology, Phenotypes, Socioeconomic

## Abstract

While deficits in episodic memory have been noted following cerebellar damage, there is a lack of research systematically exploring the socio-demographic and cognitive profiles of patients with such impairments. This study aimed to differentiate between chronic-phase cerebellar stroke patients with and without verbal episodic memory deficits, and to determine whether those with deficits exhibit distinct socio-demographic and clinical profiles, thereby identifying potential factors associated with these impairments. Data from 15 cerebellar stroke patients in the CEREBEMO cohort were analyzed, with participants categorized into two groups based on verbal episodic memory performance: deficits (*n* = 8) and no deficits (*n* = 7). Statistical analyses, including Generalized Linear Mixed Models and Chi-Squared tests, compared socio-demographic and neuropsychological variables between the groups. Significant differences were observed in socio-educational levels, with a higher proportion of patients with memory deficits at intermediate education levels. Moreover, patients with memory deficits performed worse on the Montreal Cognitive Assessment and the Trail Making Test, indicating overall lower cognitive efficiency and slower processing speed. Post-hoc analysis showed that, despite the limited sample size, our sample effectively detected a significant difference between the two groups with high statistical power. These findings highlight potential socio-educational and cognitive factors associated with memory impairments following cerebellar stroke.

## Introduction

Over the past two decades, growing evidence has highlighted the cerebellum’s role in non-motor functions, particularly cognition [1] and emotion [2]. Cerebellar lesions in clinical populations have been shown to affect notably executive function, visual processing, linguistic skills and affect regulation. These deficits are grouped under cerebellar cognitive affective syndrome (CCAS) [3], a broad concept encompassing diverse neuropsychological impairments. In this context, a recent epidemiological study estimated that 60.30% of patients experience neuropsychological deficits during the subacute phase following a cerebellar stroke, with the prevalence dropping to 51.20% in the chronic phase [4]. While tools (e.g., The cerebellar cognitive affective syndrome scale for CCAS [5]) have been developed to assess CCAS, the factors predicting or protecting against these deficits remain unclear. Among the cognitive functions affected, episodic memory has been shown to be particularly vulnerable to cerebellar damage [4], with deficits persisting into the chronic phase [4]. However, no studies have yet systematically characterized the sociodemographic and cognitive profiles of patients with cerebellar damage in relation to their verbal episodic memory deficits.

In this context, the aim of the present exploratory study was twofold. First, to differentiate, using a neuropsychological test assessing verbal episodic memory, between patients in the chronic phase following a cerebellar stroke who exhibit verbal episodic memory deficits and those who do not. Second, to determine whether patients with verbal episodic memory deficits display distinct socio-demographic and clinical profiles compared to those without such deficits, thereby identifying potential factors associated with these episodic memory impairments.

## Methodology

### Participants

As part of this exploratory study, we extracted data from the CEREBEMO cohort, a project conducted at the University of Geneva that evaluated the effects of cerebellar stroke on emotion recognition [6]. In this context, participants also underwent a neuropsychological assessment. From this cohort, we selected sociodemographic (e.g., age; gender; educational level [Level 1 is equivalent to the compulsory Swiss scholarship (< 11 years of study); level 2 is equivalent to a vocational diploma (11–12 years of study); level 3 is equivalent to Matura level and higher education (> 12 years of study)] and clinical data from 15 patients who had experienced a cerebellar stroke and for whom performance in verbal episodic memory was available. Moreover, the following exclusion criterions were applied: (i) brainstem or occipital lesion; (ii) one or more other brain lesions; (iii) diffuse and extensive white-matter disease; (iv) other degenerative or inflammatory brain disease; (v) confusion or dementia; (vi) major psychiatric disease; (vii) the wearing of hearing aids or a history of tinnitus or a hearing impairment; (viii) age below 18 years; (ix) major language comprehension deficits precluding reliable testing. These patients were categorized based on their performance in a verbal episodic memory test, the 16-item free/cued recall (in French, épreuve de rappel libre/rappel indicé à 16 items; RL/RI-16 [7]. A test assessing verbal episodic memory, consisting of an encoding phase followed by three immediate free recalls, three indexed recalls (interspersed with a reverse counting task), a delayed free recall, and a 20-minute delayed indexed recall. The patients were evaluated based on standardized norms [adjusted for age, gender and socio-cultural level] and a conservative clinical cut-off (< 5th percentile; Z-score < -1.60). Among the 15 patients, 8 exhibited deficits in at least two sub-scores, while 7 showed no deficits. This allowed us to divide the group into two subgroups: (i) patients with memory deficits (*n* = 8) and (ii) patients without memory deficits (*n* = 7). In addition to the verbal episodic memory assessment, all participants underwent a neuropsychological evaluation, including an assessment of global cognitive impairment (MoCA: Montreal Cognitive Assessment scale [8]), global executive functioning (Frontal Assessment Battery [9]), as well as executive functions, especially, inhibition (Stroop [10]), mental flexibility (TMT: Trail Making Test [11]) and verbal fluency. Finally, self-reported psychiatric symptoms as following: depression with BDI-II: Beck Depression Inventory II [12]; apathy with the AES: Apathy Evaluation Scale [13]; alexithymia with the TAS-20: Toronto Alexithymia Scale [14].

### Statistical Analysis

Given the non-parametric nature of our data, we conducted appropriate statistical analyses to evaluate the differences between the two patient subgroups. First, socio-demographic variables were analyzed using Generalized Linear Mixed Models (GLMM) of the GAMMA type for continuous variables (age, days since stroke) and Chi-Squared (*X²*) tests for categorical variables (gender, lesion lateralization, socio-educational level). Next, GAMMA and multiple-type GLMM analyses were performed to examine neuropsychological scores and psychiatric questionnaire results as the primary variables. To control for potential confounding effects, socio-demographic or clinical variables that were found to be significantly different between groups were included as covariates (random effects). This allowed us to isolate their influence and focus specifically on the neuropsychological and psychiatric outcomes of interest. Benjamini-Hochberg False Discovery Rate (FDR) type corrections were carried out.

## Results

### Sociodemographic and Clinical Variables

The analyses showed a significant difference in socio-educational level between patients with and without memory deficits, with a higher proportion of patients at the intermediate level (Level 2) in the memory deficit group, suggesting a lower overall socio-educational level in this subgroup. No significant differences were found between patient groups in terms of age, gender, lesion hemispheric localization, or time since stroke.

To further investigate the specific effect of other variables (see below), the socio-educational level was subsequently included in the analysis. This step was necessary to avoid potential confounding effects, as memory deficits were the primary discriminating variable.

**Table 1:** Comparison of demographic and clinical characteristics between cerebellar stroke patients with and without verbal episodic memory deficits (General Linear Mixed Models GAMMA type for continuous variables (age, days since stroke) and Chi-Squared (X²) tests for categorical variables (gender, lesion lateralization, socio-educational level)

	Cerebellar patients *without* memory deficits (*n* = 7)	Cerebellar patients *with* memory deficits (*n* = 8)	Stat. and *p*. values
Age in years (mean ± SD)	59.57 ± 14.62	61.38 ± 14.39	*B*= -0.232, *p* =.816
Gender (% women)	14.29%	37.50%	*X*^*2*^ = 1.03, *p* =.310
Socio-cultural level (% levels)	Level 1:0% Level 2: 0% Level 3: 100%	Level 1:0% Level 2: 75% Level 3: 25%	***X***^***2***^ **= 8.75**, ***p*** =.003**
Hemispheric localization of the cerebellar lesion (L/R/Bilateral)	(1/4/2)	(3/5/0)	*B* = -0.70, *p* =.526
Time since stroke in days (mean ± SD)	370.00 ± 270.21	773.75 (± 801.56)	*B* = -0.985, *p* =.336

Legend: L: left; R: right; SD: standard deviation

** Significant after Benjamini-Hochberg FDR correction

### Neuropsychological Performances and Self-Reported Psychiatric Symptoms

#### Neuropsychological Performances

**Fig. 1:**
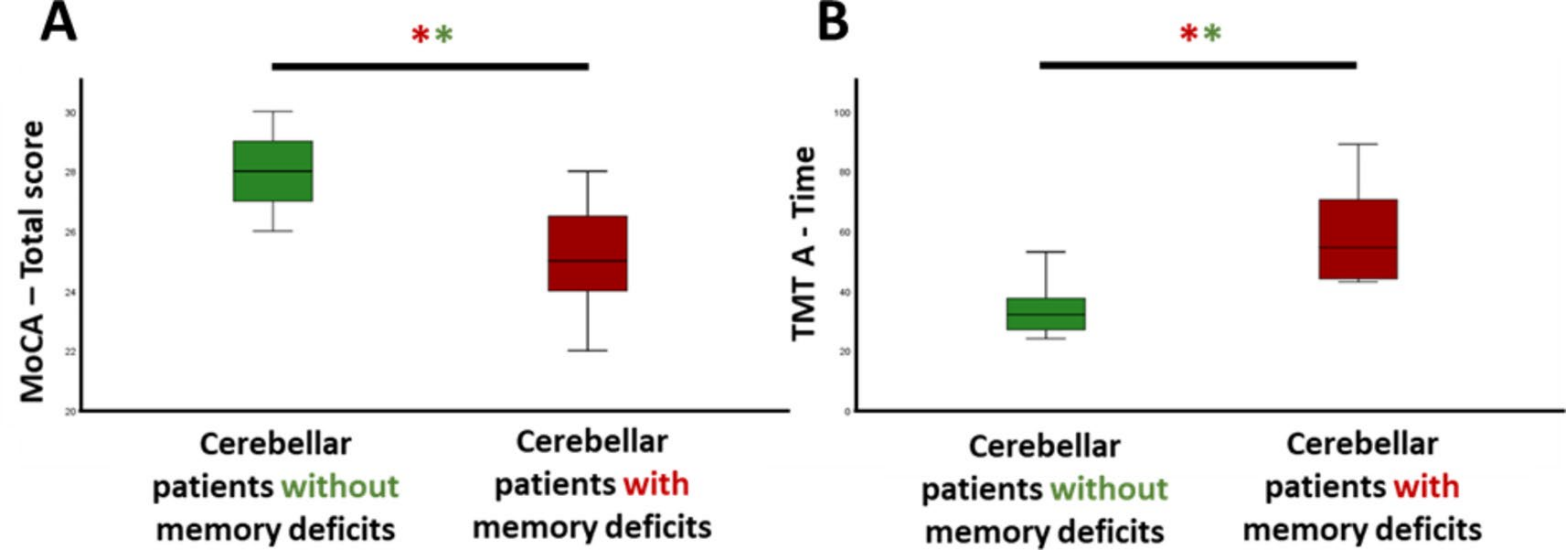
Significant differences between the group of cerebellar patients with episodic memory deficits and the group of cerebellar patients without episodic memory deficits (Benjamini Hochberg FDR correction applied). (**A**) Patients with memory deficits showed significantly lower scores in global cognitive efficiency, as measured by the MoCA, compared to those without memory deficits. (**B**) Additionally, patients with memory deficits had significantly longer completion times on the mental flexibility task, as assessed by the Trail Making Test, compared to those without memory deficits. Legend: MoCA: Montreal Cognitive Assessment [8]; TMT: Trail Making Test [11]. ** Significant after Benjamini-Hochberg FDR correction

Significant differences were found between the two patient subgroups for the MoCA Total score and the TMT A Time score, with poorer performance for both measures observed in cerebellar patients with memory deficits. All other comparisons were non-significant after multiple comparison correction (see Table 2; Fig. 1).

#### Self-Reported Psychiatric Symptoms

No significant differences were found between cerebellar patients with and without memory deficits (*p* >.088).

**Table 2:** Comparison of neuropsychological and psychiatric data between cerebellar stroke patients with and without verbal episodic memory deficits, based on the following statistical analysis: GAMMA for continuous and Multinomial for categorical Generalized Linear Mixed Models

	Cerebellar patients without memory deficits (*n* = 7)	Cerebellar patients with memory deficits (*n* = 8)	Stat. and *p*. values
MoCA – Total score	28.00 ± 1.53	25.13 ± 1.96	***B*= -3.07**, ***p*** =.009**
* Executive and visuospatial*	4.86 ± 0.38	4.00 ± 1.41	*B* = -0.01, *p* =.999
* Denomination*	3.00 ± 0.00	2.87 ± 0.35	*B* = -0.63, *p* =.539
* Attention*	6.00 ± 0.00	5.38 ± 1.06	*B* = -0.01, *p* =.999
* Language*	2.71 ± 0.49	2.50 ± 0.54	*B* = -0.78, *p* =.449
* Abstraction*	2.00 ± 0.00	1.63 ± 0.52	*B* = -1.26, *p* =.228
* Recall*	3.71 ± 1.25	2.38 ± 1.18	*B* = -0.05, *p* =.997
* Orientation*	5.86 ± 0.38	6.00 ± 0.00	*B* = -0.73, *p* =.480
FAB – Total score	17.14 ± 0.90	14.00 ± 2.73	*B* = -2.64, *p* =.020
* Similarities*	3.00 ± 0.00	2.75 ± 0.46	*B* = -1.01, *p* =.332
* Lexical Fluency*	2.71 ± 0.49	2.63 ± 0.52	*B* = -0.34, *p* =.739
* Motor Sequences*	2.71 ± 0.49	1.88 ± 0.99	*B* = -1.29, *p* =.240
* Flexibility*	2.71 ± 0.49	2.63 ± 0.52	*B* = -0.34, *p* =.739
* Go/No-Go*	3.00 ± 0.00	1.13 ± 1.36	*B* = -0.01, *p* =.999
* Grip*	3.00 ± 0.00	3.00 ± 0.00	*B* = 0, *p* = 1
Fluency 2’ (Categorical)	29.14 ± 7.80	22.75 ± 6.86	*B* = 1.66, *p* =.122
Fluency 2’ (Literal)	20.80 ± 4.32	15.33 ± 9.09	*B* = 1.36, *p* =.207
Stroop – Denomination time (sec.)	67.67 ± 14.94	73.38 ± 17.26	*B* = 1.36, *p* =.207
Stroop – Lecture time (sec.)	47.83 ± 13.96	52.00 ± 8.07	*B* = -0.86, *p* =.404
Stroop – Interference (sec.)	119.33 ± 23.89	151.00 ± 38.15	*B* = -1.64, *p* =.127
Stroop – Inhibition	51.67 ± 27.64	77.63 ± 23.22	*B* = 1.968, *p* =.073
TMT (A) – Time in seconds	34.00 ± 10.00	58.75 ± 16.71	***B*** **= 3.68**, ***p*** =.003**
TMT (B) – Time in second	81.00 ± 36.72	118.50 ± 21.57	*B* = -2.74, *p* =.017
TMT B/A	47.00 ± 31.74	59.75 ± 29. 57	*B* = -1.10, *p* =.293
Apathy (AES) – Total score	0.00 ± 0.00	6.75 ± 9.63	*B* = -1.84, *p* =.088
Depression (BDI – II) – Total score	10.20 ± 4.66	14.00 ± 6.68	*B* = -0.95, *p* =.365
* Cognitive subscore*	7.20 ± 1.79	9.25 ± 5.97	*B* = -0.23, *p* =.822
* Affective subscore*	2.40 ± 2.61	4.75 ± 5.75	*B* = -0.69, *p* =.510
Alexithymia (TAS-20) – Total score	57.40 ± 12.36	55.38 ± 15.92	*B* = -0.31, *p* =.763
* Description-feeling*	17.60 ± 4.45	14.75 ± 4.03	*B* = -1.08, *p* =.302
* Open-mindedness*	21.20 ± 4.82	20.75 ± 9.05	*B* = -0.33, *p* =.750
* Identification feeling*	18.60 ± 5.46	19.88 ± 5.84	*B* = 0.42, *p* =.684

Legend: AES: Apathy Evaluation Scale [13]; BDI-II: Beck Depression Inventory II [12]; FAB: Frontal Assessment Battery [9]; MoCA: Montreal Cognitive Assessment [8]; Stroop [10]; TAS-20: Toronto Alexithymia Scale [14]; TMT: Trail Making Test [11]

** Significant after Benjamini-Hochberg FDR correction

## Post-Hoc Power Analyses

Given the exploratory nature of our study and the small sample size, we performed post-hoc power analyses to determine the power of our results on the scores that were significantly different between the groups after correction: MoCA and TMT A. Post-hoc power analyses equations modelled on “https://clincalc.com/stats/power.aspx” were performed. A statistical power of 88.9% for MoCA Total-Score and 94.2% for TMT A score were found.

## Discussion

This exploratory study identified a significant difference in socio-educational levels between chronic cerebellar stroke patients with and without verbal episodic memory deficits, with a higher proportion of individuals at intermediate socio-educational levels in the memory deficit group, suggesting a potential link between lower educational status and memory impairments. Additionally, patients with verbal episodic memory deficits demonstrated poorer overall cognitive performance and reduced processing speed, even when accounting for socio-educational factors. These exploratory results are significant for several reasons.

Firstly, these findings emphasize the importance of assessing chronic cerebellar stroke patients based on both their lesions and cognitive symptoms, suggesting distinct patient phenotypes with specific neuropsychological profiles. In this case, memory impairments were linked to overall cognitive decline and slower processing speed. Interestingly, the significant result for overall cognitive decline (measured with MoCA) was not driven by the memory subscore, suggesting an accumulation of different alterations at different subscores. Other clinical phenotypes may also exist, potentially encompassing psychiatric [2], affective [2] or sensori-motor [3] symptoms, highlighting the heterogeneity of the consequences of cerebellar damage. Recognizing these distinct profiles may allow for a more nuanced understanding of how cerebellar damage impacts cognitive functioning, thereby facilitating tailored rehabilitation strategies.

Secondly, the findings highlight the potential influence of socio-educational level in cerebellar stroke. These results align with previous research suggesting that socio-educational level is an important factor in the resilience to cognitive disorders, particularly in the context of episodic memory, but this is also in line with an emerging literature indicating that socio-cultural factors can influence cerebellar structure, including findings that lower socio-economic status is associated with reduced cerebellar volume [14]. This suggests that the cerebellum may be particularly sensitive to environmental influences [15]. This observation is particularly noteworthy given that high socio-economic status is widely recognized as a protective factor against the development and chronicity of neuropsychological deficits [16]. Our exploratory findings thus imply that the cerebellum, being sensitive to socio-economic conditions, may be more susceptible to memory deficits in patients who have experienced a cerebellar stroke and possess low socio-economic status.

The main limitations of this study include the small sample size (15 patients), which restricts the generalizability of the findings, and its exploratory nature, necessitating further research to confirm the observed associations between socio-educational status and cognitive deficits following cerebellar stroke. However, a post-hoc power analysis indicates that, despite the small sample size, the inter-group differences show sufficient statistical power, supporting potential relevance beyond this sample. An additional limitation is that we observed an effect only on the total MoCA score, but not on the individual subtests. Interpreting this as an indication of altered cognitive efficiency could be an over-interpretation. Future studies using a variety of global scales will be needed to replicate these findings.

Overall, these findings emphasize the impact of environmental factors on cerebellar function and cognitive health. They underscore the importance of exploring symptom assessment to enhance clinicians’ ability to predict recovery trajectories and optimize treatment plans, ultimately leading to improved patient outcomes. Future studies would benefit from incorporating longitudinal data to better understand the progression of cognitive deficits over time and provide deeper insights into the long-term impact of cerebellar stroke on cognitive and affective functions.

## Data Availability

Data are available on request from JA.P.
